# Comprehensive Axillary Management of Clinically Node-Positive (cN+) Breast Cancer Patients: A Narrative Review on Neoadjuvant Chemotherapy

**DOI:** 10.3390/cancers16193354

**Published:** 2024-09-30

**Authors:** Calogero Cipolla, Vittorio Gebbia, Eleonora D’Agati, Martina Greco, Chiara Mesi, Giuseppa Scandurra, Maria Rosaria Valerio

**Affiliations:** 1 Department of Precision Medicine in Medical, Surgical and Critical Care (Me.Pre.C.C.), University of Palermo, 90100 Palermo, Italy; calogero.cipolla@unipa.it (C.C.); dagatieleonora@gmail.com (E.D.); doc.martinagreco@gmail.com (M.G.); chiaramesi95@gmail.com (C.M.); mariarosaria.valerio@unipa.it (M.R.V.); 2 Breast Unit, AOUP Policlinico Paolo Giaccone, 90100 Palermo, Italy; 3 Department of Medicine and Surgery, Kore University of Enna, 94100 Enna, Italy; giuseppa.scandurra@unikore.it; 4 Medical Oncology Unit, Cdc Torina, 90100 Palermo, Italy; 5 Medical Oncology Unit, AOUP Policlinico Paolo Giaccone, 90100 Palermo, Italy; 6 Medical Oncology Unit, Ospedale Cannizzaro, 95126 Catania, Italy

**Keywords:** breast cancer, multidisciplinary management, neoadjuvant chemotherapy, node-positive disease

## Abstract

**Simple Summary:**

Axillary management in breast cancer has undergone significant changes over the past decades, especially with the introduction of neoadjuvant chemotherapy (NACT). NACT aims to shrink tumors before surgery, allowing for less invasive axillary approaches such as sentinel lymph node biopsy (SLNB) and targeted axillary dissection (TAD). These techniques help reduce the need for axillary lymph node dissection (ALND), which is associated with higher risks of complications like lymphedema. However, patient selection for these procedures depends on factors such as tumor biology, response to NACT, and the extent of nodal disease. This review discusses the latest evidence supporting de-escalation strategies in axillary surgery and highlights ongoing research that aims to further refine the selection criteria for these approaches. Multidisciplinary collaboration remains key to implementing personalized treatments that optimize patient outcomes while minimizing surgical morbidity.

**Abstract:**

Background. In breast cancer (BC) patients, axillary management has undergone major improvements over the last few years, and efforts to identify the optimal strategy for the management of axillary surgery are still ongoing. Methods. In current clinical practice, women with clinically node-positive (cN+) BC usually receive neoadjuvant chemotherapy (NACT) with the aim of reducing the extent of primary disease and, thus, allowing for axillary-conservative surgery. Remarkably, after NACT, up to one out of three patients achieves an axillary pathologic complete response, which, in turn, is associated with a more favorable prognosis than residual axillary disease. However, NACT is not without drawbacks, as NACT-associated inflammation can damage lymphatic vessels. Furthermore, varying degrees of response may occur in the axillary lymph nodes, increasing the false negative rate for sentinel biopsy. Results. At present, there is no consensus on the optimal approach in patients with cN+ BC undergoing NACT, although multidisciplinary management seems to be recommended. Conclusions. This narrative review provides a comprehensive overview of axillary management in cN+ BC patients undergoing NACT. It uses a multidisciplinary approach that encompasses the oncological management perspectives, as well as surgical and chemotherapeutic viewpoints.

## 1. Introduction

Axillary management in breast cancer (BC) has undergone fundamental changes over the last few decades [1,2,3,4]. In particular, a significant reduction in axillary lymph node dissection (ALND) use in clinical practice has been reported given the substantial morbidity associated with this approach, such as shoulder stiffness, arm lymphedema, numbness, paresthesia, chronic pain, limited movement, lymphangitis, and tissue fibrosis [1,5,6,7,8,9,10]. Efforts to identify the optimal strategy for the management of axillary surgery continue to evolve.

In current clinical practice, women with clinically node-positive (cN+) BC usually receive neoadjuvant chemotherapy (NACT) [9,11,12]. In particular, in locoregionally advanced BC, NACT helps reduce the extension of primary disease, thus permitting more axillary conservative surgery than ALND and sparing the possible complications of this approach [12]. Remarkably, after NACT, a substantial proportion of patients, up to one out of three, achieve an axillary pathologic complete response (pCR), which, in turn, is associated with an improved prognosis compared with residual axillary disease [13].

Patients suitable for NACT are heterogeneous, and many NACT schemes have been proposed based on the molecular characteristics of the underlying disease (Table 1) [12,14]. NACT has its drawbacks, as NACT-associated inflammation can damage lymphatic vessels and induce an anatomical modification of the lymphatic system. Furthermore, a different degree of response may occur in the axillary lymph nodes (LNs), increasing the false negative rate (FNR) for sentinel biopsy [12].

A consensus has not been reached on the optimal approach in patients with cN+ BC undergoing NACT, considering the various oncological, surgical, and chemotherapeutic perspectives [15,16,17,18]. However, multidisciplinary management seems to be the most recommended strategy [19,20,21]. Based on the available evidence and the authors’ experiences, this narrative review aims to provide a comprehensive overview of axillary management in cN+ BC patients undergoing NACT. It uses a multidisciplinary approach that encompasses oncological management perspectives, as well as surgical and chemotherapeutic viewpoints. This review focuses on the evaluation before NACT, NACT treatment by the molecular subtype, the assessment of NACT outcomes, and supporting strategies.

## 2. Evaluation before Neoadjuvant Therapy

ALND can be avoided in cN+ patients receiving NACT if three or more negative sentinel LNs (SLNs) are reported [22,23,24]. A summary of recent notable studies is provided in Table 2.

The study by Montagna et al. evaluated how often cN+ patients avoid ALND by receiving NACT in a prospectively collected database of 630 patients [22]. Of them, 573 (91%) were converted to clinically node-negative (cN0) and underwent SLN biopsy (SLNB), and 531 (93%) showed ≥3 excised SLNs. Lymphovascular invasion (LVI) and increased body mass index were associated with a failure to identify ≥3 SLNs. The pCR was reported for 255/573 (46%) patients, and 237 (41%) had adequate mapping. Factors associated with ALND avoidance were high grade, receptor status, and LVI. Overall, similar findings were reported by Cipolla et al. in two consecutive studies [23,24].

The assessment of axillary status after NACT is crucial for selecting appropriate treatment decisions (Table 3) [25,26]. Indeed, the presence of LN metastases may suggest performing ALND. Axillary status is assessed by mammography, computed tomography (CT), and magnetic resonance imaging (MRI), but ultrasonography (US) remains the gold standard, although the negative predictive value of US is higher in overweight patients [25,26,27].

Fine-needle aspiration (FNA) may enhance the performance of the US, as suggested by Hotton et al. in a retrospective study of 292 patients [28]. Of them, 88 (30.1%) had a suspicious LN on US and underwent FNA: 53 tested positive for axillary LN involvement (60.2%), and among the 35 patients who tested negative, 15 had axillary metastatic involvement. Overall, the performance of US plus FNA was better than that of US alone; luminal A subgroup, axillary involvement of <2 LN+, or nodal tumor <7 mm were independent factors of FNR.

The accuracy of the evaluation can be further improved by marking positive LNs using a dual tracer [29,30]. In a systematic review of 24 studies, the combination of radioisotope and blue dye showed an overall higher identification rate than radioisotope alone (OR = 2.03; 95% CI: 1.53–2.69; *p* < 0.05), but this advantage was not reported for patients receiving NACT (OR = 1.64; 95% CI: 0.82–3.27) [29]. More recently, in a retrospective study of 73 cN+ patients receiving NACT who underwent the placement of a clip in the positive LN before treatment, targeted axillary dissection (TAD) was more accurate than SLN alone, suggesting changes in management in a remarkable proportion (10%) of patients [30].

## 3. Neoadjuvant Therapy in Different Molecular Subtypes

Tumor biology is a major predictor of pCR in BC patients undergoing NACT, with remarkable importance for therapy selection [12,31,32]. Indeed, a pooled analysis of 33 studies published in 2021, comprising 57,351 patients, showed a pCR rate of 60% for HR−/HER+ BC, followed by 59% for HER+ disease, 48% for triple-negative breast cancer (TNBC), 45% for HR+/HER2+, 35% for luminal B disease, 18% for HR+/HER2+, and 13% for luminal A disease. Overall, similar findings were reported a year later by Wolf et al., who employed a different classification of BC subtypes, considering several biomarkers [32]. We report here information on pCR rates from specific studies on various BC subtypes.

### 3.1. Luminal A and B Subtypes

In the context of NACT, luminal BC, particularly the luminal A subtype, has demonstrated lower sensitivity to cytotoxic agents compared with more aggressive subtypes such as HER2-positive or triple-negative breast cancer [33]. This underscores the challenges in achieving significant tumor reduction through conventional chemotherapeutic approaches in patients with luminal BC [34]. Historically, clinical trials have struggled to optimize chemotherapy regimens for luminal breast cancer due to the inherent chemoresistance [34].

Collins et al. investigated factors associated with histopathologic response and oncologic outcome following NACT in 114 women with luminal A disease [33]; pCR was reached in 7.9% of patients, ypN0 in 25.5%, and downstaging in 33.6%. Tumor grade was an independent predictor of pCR (*p* = 0.039), while PR score predicted ypN0 (*p* = 0.017) and downstaging (*p* = 0.029). The 5-year invasive disease-free survival (DFS) rate was 68.5 ± 4.7%, and the overall survival (OS) rate was 77.7 ± 4.3%. On the other hand, luminal B BC has a worse prognosis than luminal A BC and presented a lower sensitivity to chemotherapy than non-luminal subtypes [34,35]. In an exploratory analysis of the Gruppo Italiano Mammella 2 (GIM2) randomized trial, Conte et al. investigated the efficacy of dose-dense NACT (anthracyclines followed by paclitaxel) compared with standard interval NACT, according to luminal-like subtypes [34]. Among the 2003 patients enrolled in the GIM2 trial, 412 had luminal A disease and 638 luminal B disease. At a median follow-up of 7.9 years, DFS was 80.8% (95% CI: 76.4–84.5) in luminal A disease and 70.5% (95% CI: 66.5–74.2) in luminal B disease; the corresponding figures for OS were 91.6% (88.2–94.1) and 85.1% (81.7–87.9), respectively. Overall, patients with luminal B disease appeared to benefit more from the dose-dense NACT than those with luminal A BC, both in terms of DFS (HR = 0.72; 95% CI: 0.54–0.96) and OS (HR = 0.6; 95% CI: 0.40–0.94), compared with the luminal A-like cohort (HR for DFS = 0.89; 95% CI: 0.59–1.33; HR for OS = 0.83; 95% CI: 0.45–1.54). In a retrospective study of 205 luminal-like, node-positive breast cancer patients who underwent NACT, Barbieri et al. demonstrated a low pCR rate in both the primary breast tumor and axillary lymph nodes [36]. Furthermore, the study found no difference in DFS or OS between patients who received ALND and those who underwent SLNB alone.

The randomized, phase II Neo-CheckRay trial is evaluating stereotactic body radiation therapy to the primary BC in combination with the adenosine pathway inhibitor oleclumab to improve the response to NACT in patients with luminal B disease [35]. The preliminary results of the safety run-in analysis, obtained in six patients over a 2-year follow-up, suggest that this treatment combination is worth further investigation given the lack of major adverse events and overall favorable cosmetic outcomes.

### 3.2. HER2+ Subtype

Numerous studies in the HER2+ setting have supported the efficacy of NACT in combination with targeted agent-containing regimens in inducing downstaging [31,37].

In the pivotal phase III NeoALTTO randomized trial, published in 2012, Baselga et al. showed that dual inhibition of HER2 by administering lapatinib and trastuzumab might be effective in the neoadjuvant setting [38]. These findings were mirrored by those reported in the CHER-LOB trial, which documented a pCR rate of 46.7% with the combination of these two agents [39]. Other trials have emphasized the role of combination therapy [40,41,42,43,44,45,46,47,48].

### 3.3. Triple-Negative Breast Cancer

The treatment of TNBC is particularly challenging, and a high proportion of patients experience recurrence within 5 years of completing neoadjuvant therapy [49]. Given the lack of targetable receptors, adding immunotherapy to NACT seems a suitable strategy [50,51]. The I-SPY2 trial showed a 60% pCR rate for the pembrolizumab plus paclitaxel cohort, as compared with a 22% pCR rate with paclitaxel alone, which was overall predictive of long-term outcomes [52]. The pivotal KEYNOTE-522 trial investigated whether adding pembrolizumab to paclitaxel plus carboplatin NACT could increase the pCR rate in patients with TNBC [53]. At the first interim analysis, the pCR rate was significantly higher with the addition of immunotherapy than NACT alone (64.8% vs. 51.2%; *p* < 0.001). These data were undoubtedly practice-changing and led to the approval of this strategy; however, it is essential to remark that the G ≥ 3 adverse event rate was not negligible (78% with pembrolizumab plus NACT vs. 73% vs. NACT alone).

In the phase II GEPAR-NUEVO study, the addition of durvalumab to NACT achieved a nonsignificant increase (9%) in the pCR rate compared with NACT alone; this effect was observed only in patients who received durvalumab before NACT [54]. The phase III IMpassion031 trial compared atezolizumab plus nab-paclitaxel followed by doxorubicin/cyclophosphamide in the neoadjuvant setting vs. NACT alone [55]. The rate of pCR was 58% in the atezolizumab plus NACT group and 41% in the NACT-only group (*p* = 0.0044); this difference was even more evident when considering patients with programmed death-ligand 1 (PD-L1)-positive disease. However, these findings were challenged by another study by Gianni et al., which showed that the addition of atezolizumab to NACT based on nab-paclitaxel and carboplatin did not increase the pCR rate, although the effect of this strategy in PD-L1 disease was still reported [56]. Overall, these data favor using an immunotherapy-based preoperative strategy in TNBC, although some aspects deserve a more in-depth analysis.

## 4. Evaluation after Neoadjuvant Therapy

### 4.1. Assessment of pCR by Imaging

The achievement of pCR after NACT plays a pivotal role in guiding the subsequent management of patients [9,57]. Therefore, accurate assessment of pCR is of the utmost importance.

Gu et al. performed a meta-analysis of 62 studies to evaluate the accuracy of contrast-enhanced and diffusion-weighted MRI (CE-MRI and DW-MRI) in identifying the response to NACT [57]. CE-MRI showed high specificity, while DW-MRI had high sensitivity in predicting pCR after NACT. Moreover, CE-MRI was more accurate than ultrasound (US) or mammography. The authors concluded that the combined use of CE-MRI and positron emission tomography/computed tomography (PET/CT) or DW-MRI could enable a precise assessment of patients receiving NACT in the neoadjuvant setting.

### 4.2. Axillary Management

Recommendations for regional irradiation after NACT must still be based on solid evidence [58,59]. Heidinger et al. recently reviewed the surgical and radiotherapy (RT)-based axillary management of patients with cN+ BC [60]. While the reader is referred to that manuscript for a more comprehensive source of information on the topic, we provide some specific considerations here. In 2021, Barrio et al. evaluated nodal recurrence rates in 610 cN+ patients receiving NACT, followed by a negative SLNB and no further axillary surgery [61]. Among them, 555 (91%) converted to cN0 disease and underwent SLNB; 234 (42%) had ≥3 negative SLNs and underwent SLNB alone. Of these, 205 (88%) received adjuvant RT, and 164 (70%) also received nodal RT. At 40-month follow-up, there were no nodal recurrences in patients who received RT.

TAD consists of removing biopsy-proven positive axillary nodes, which are marked (marked LNB) before NACT in addition to SLNB [62]. Although TAD has shown high identification rates (97%) and a low false negative rate (7%), there remain ongoing discussions regarding its long-term implications for prognosis [62]. In a systematic review of nine studies, including 366 patients, Swarnkar et al. compared and determined the FNR of TAD with complete ALND [62]. Overall, TAD was associated with an FNR of 5.2%, similar to marked LNB alone, thus suggesting that SLNB can be safely omitted from TAD.

Supporting these findings, Bartles et al. recently reported the 10-year analysis of axillary recurrence rate (ARR), OS, and DFS from the large EORTC 10981-22023 AMAROS trial [8]. A total of 4806 patients underwent SLNB; 1425 were cN+ and randomly assigned to either ALND or RT. The 10-year ARR was 0.93% after ALND and 1.82% after adjuvant RT (HR = 1.71; 95% CI: 0.67–4.39). No differences were found in OS or DFS, and the quality of life (QoL) was similar. However, ALND was associated with a higher lymphedema rate (24.5% vs. 11.9%; *p* < 0.001). The authors of this study concluded that adjuvant RT may be preferred over ALND for cN+ patients due to reduced locoregional morbidity.

Recent studies have shown promising results in the de-escalation of axillary surgery. Nijveldt et al. reported an 84% reduction in ALND procedures following the implementation of TAD and noted that 18% of patients did not receive adjuvant axillary radiotherapy [63]. Additionally, Montagna et al. found no significant difference in axillary recurrence rates between patients treated with TAD versus SLNB alone, supporting the omission of ALND in carefully selected patients [64]. Furthermore, TAD alone may offer comparable survival outcomes and recurrence rates to TAD with ALND, particularly in patients showing good clinical response to NACT and at least three targeted LNs [65]. Moreover, for women with cN1 breast cancer who convert to ypN0 after NACT and undergo breast-conserving surgery with SLNB, there is growing evidence that more extensive regional nodal irradiation may not provide additional long-term survival benefits. This approach is currently being investigated in the NSABP B-51 trial [66]. Therefore, TAD may represent an alternative to extensive surgical procedures in specific patient populations, further supporting the de-escalation of axillary management. However, the successful implementation of this technique requires close collaboration between breast radiologists, surgeons, and pathologists, and further research is needed to standardize selection criteria and confirm its oncological safety.

Breast-conserving therapy (BCT) consists of breast-conserving surgery and subsequent RT [67]. This approach is still underused worldwide, especially in non-Western countries, but it is recommended by international guidelines [67]. Possible solutions for broader use of BCT after NACT include increased multidisciplinary management, optimized treatment counseling, and easier access to this approach. Patients should be regularly monitored for axillary status during follow-up visits.

#### The Management of Micrometastatic Disease

Residual micrometastatic disease (SLNmi) in the axilla following NACT is a negative prognostic factor, also associated with additional non-sentinel lymph node (non-SLN) metastases [68]. In contrast, residual nodal micrometastasis (ypN1mi) does not influence prognosis compared to ypN0, suggesting that additional ALND may be warranted to confirm the axillary nodal status in patients with SLNmi [69].

However, caution should be exercised in changing clinical practice based on these findings, as the omission of ALND may be safe in patients with isolated tumor cells [60]. Prospective validation of these approaches is currently being evaluated in trials such as the NEONOD2 trial, which aims to assess the safety of omitting axillary surgery in patients with SLN micrometastasis after NACT [70].

## 5. Supportive and Psychological Care

The diagnosis of BC and the therapeutic sequelae represent formidable physical and emotional distress for both patients and their families, which may hamper the QoL, therapeutic strategy, and ultimate clinical results. It is widely recognized that women with BC require dedicated care to limit possible complications and manage the psychological issues that occur during neoadjuvant treatment [71].

A Cochrane review published in 2016 investigated the effect of aerobic exercise on treatment-related adverse events during NACT [72]. A total of 32 studies involving 2626 patients were considered. Physical exercise during NACT improved physical fitness (SMD = 0.42; moderate-quality evidence) and reduced fatigue (SMD = −0.28; moderate-quality evidence). Physical training was also associated with modest improvements in cancer site-specific QoL and cognitive function but not with improvements in cancer-specific QoL or depression. Efforts are ongoing to further investigate the role of physical exercise in preventing and managing adverse events during NACT [73].

Despite the undisputed importance of psychological and emotional well-being during treatment, only a few studies have assessed patients’ experiences before and during NACT. In a recent survey, Omari et al. determined the prevalence of psychological distress before NACT in 209 patients [74]. The prevalence of depression was 59.6%, that of anxiety was 47.8%, and the prevalence of psychological distress was 65.1%. Depression and anxiety were associated with younger age (<50 years), while psychological distress was associated with chronic illness and LN status. In another study on 53 patients, Tschuschke et al. investigated the impact of psychological factors on women undergoing NACT [75]. The authors of this study remarked that women undergoing NACT have to deal with emotional shock due to the cancer diagnosis and the fact that the malignant tumor will be removed only after completing chemotherapy. Women were evaluated before starting NACT and immediately after completing treatment but before surgery. Patients were also followed up to 5.5 years after NACT. Overall, poor coping behavior (resignation, no attempt to seek social support) was associated with an increased risk of recurrence or developing another malignancy. On these bases, psychological screening immediately after the diagnosis and before any oncological treatment is recommended to identify patients needing additional psycho-oncological support at an early stage.

## 6. Concluding Remarks

Axillary management in breast cancer has seen substantial advancements, particularly with the growing emphasis on de-escalating surgical interventions to reduce morbidity while maintaining oncological safety. NACT has played a pivotal role in these changes by allowing for tumor downstaging and facilitating less invasive axillary approaches.

The selection of the axillary management strategy is strongly dependent on clinical and biomolecular characteristics, as various subtypes of BC differ significantly. In patients with more aggressive subtypes, such as TNBC or HER2+ disease, the likelihood of achieving a pCR is higher, which may justify the use of less invasive procedures like SLNB or TAD. In contrast, luminal-like subtypes, which tend to have lower pCR rates, may require more extensive axillary evaluation or consideration of ALND, particularly in cases with residual nodal disease post-NACT.

Clinical response to NACT, nodal burden, and patient comorbidities also play a critical role in tailoring treatment. For example, patients with low-volume nodal disease who achieve nodal downstaging (ypN0) may be candidates for SLNB or TAD alone, while those with residual micrometastases or macrometastases (ypN1) may benefit from ALND or regional nodal irradiation (RNI). Individualizing treatment based on these criteria ensures that the approach is optimized for each patient, balancing oncological safety with the minimization of morbidity.

The shift toward less invasive surgical techniques, supported by emerging evidence from randomized trials, such as the NSABP B-51 and ALLIANCE A011202 studies, is a significant step forward. Data from the NRG Oncology/NSABP B-51/RTOG 1304 trial, presented at the 2023 San Antonio Breast Cancer Symposium, suggest that regional nodal irradiation may be safely omitted in patients with cN1 disease who achieve a ypN0 response after NACT [76]. While the 5-year data are promising, a lower-than-expected recurrence rate has impacted the ability to perform planned statistical analyses. Nevertheless, the trial’s long-term follow-up will provide more robust data in the coming years. Additionally, the ALLIANCE A011202 trial is investigating outcomes in patients with residual nodal disease and receiving either axillary radiation therapy ART or ALND. The trial’s results, expected around 2030, will further inform the optimal approach to axillary management in this population. These trials will provide essential data to guide future decisions regarding surgical and radiation de-escalation in breast cancer patients, further refining current management protocol.

At present, there are still few indications for ALND, and real-world studies suggest safe omitting opportunities, even in cases where isolated tumor cells are discovered after NACT. Although randomized studies are difficult to conduct in this setting, well-conducted, large observational studies have the power to address this issue. We advocate that more such studies will be conducted in the future with the aim of providing clinicians with guidance grounded in clinical practice.

## Figures and Tables

**Table 1:** Common approaches to neoadjuvant therapy in patients with node-positive breast cancer according to the molecular characteristics of the disease.

Molecular Characteristics	Type of Neoadjuvant Therapy
Luminal A	Endocrine therapy
Luminal B, ER+ and/or PgR+ and HER2−	Endocrine therapy plus chemotherapy
Luminal B, HER2+	Endocrine therapy plus chemotherapy plus anti-HER2 agents
Triple-negative	Chemotherapy

Source: data taken from [12,14].

**Table 2:** Summary of recent studies on using ALND in patients with three or more negative sentinel lymph nodes.

Study	Design	Patients	Main Outcomes
Montagna et al. [22]	Observational, prospective	630 with cT1-3 disease who converted to cN0 after NACT and SLN biopsy with dual mapping	ALND was avoided in 41% of cN+ patients. Increased BMI and LVI were associated with lower rates of ≥3 SLNs.
Cipolla et al. [23]	Observational, retrospective	160 patients with cT1-3 cN+ undergoing NACT	Intraoperative SLN FNR was 38.2%, with smaller nodal volume associated with lower FNR. PPV of physical examination was 87.1%, and PPV of nodal assessment post-NACT was 68.2%.
Cipolla et al. [24]	Observational, retrospective	195 patients with positive axillary LN at diagnosis who underwent NACT	84% of cN+ patients were eligible for SLNB after NACT. ALND could be avoided in approximately 30% of cases.

ALND: axillary lymph node dissection; FNR: false negative rate; LVI: lymphovascular invasion; NACT: neoadjuvant chemotherapy; PPV: positive predictive value; SLN: sentinel lymph nodes.

**Table 3:** Lymph node imaging modalities in primary breast cancer.

Modality	Pros	Cons	Sensitivity	Specificity
PE	Accessible	Low sensitivity	30%	93%
MG	Accessible	Low sensitivity	67%	81%
US	Low cost Accessible Biopsy guidance	Operator-dependent	87%	53–97%
CT	Not recommended	Low specificity	72%	40%
MRI	Potential for LN-specific MRI contrast agents	Moderate sensitivity and specificity Limited ability to visualize the axilla	77%	90%
PET/CT	Allows for the identification of advanced axillary disease and metastatic disease	Low spatial resolution	64%	93%
PET/MRI	Improves the diagnostic performance of axillary nodal staging	Limited availability Expensive	77%	100%
SPCT/CT	Precise anatomic localization of sentinel LN	Expensive	75%	90%

CT, computed tomography; LN, lymph node; MG, mammography; MRI, magnetic resonance imaging; NA, not available; PE, physical examination; PET, positron emission tomography; SE, sensitivity; SP, specificity; SPECT/CT, single-photon emission computed tomography and computed tomography; US, ultrasonography. Source: Marino et al., 2020 [26]. Adapted from Oxford University Press under a Creative Commons (CC BY-NC-ND 4.0) licence.
